# Avermectin poisoning with panic-like symptoms: a case series

**DOI:** 10.3389/ftox.2026.1888317

**Published:** 2026-10-26

**Authors:** Shizhen Lv, Zhongchen Zhang, Guangcai Yu, Baotian Kan, Xiangdong Jian

**Affiliations:** 1 Department of Poisoning and Occupational Diseases, Emergency Medicine, Qilu Hospital of Shandong University, Cheeloo College of Medicine, Shandong University, Jinan, Shandong, China; 2 Shandong University Hospital, Shandong University, Jinan, Shandong, China; 3 First College of Clinical Medicine, Shandong University of Traditional Chinese Medicine, Jinan, Shandong, China; 4 Department of Geriatric Medicine, Department of Nursing, Qilu Hospital of Shandong University, Cheeloo College of Medicine, Shandong University, Jinan, Shandong, China

**Keywords:** acidosis, avermectin poisoning, hypotension, intramuscular injection, panic-like symptoms, psychological care, respiratory failure

## Abstract

**Background:**

High doses of avermectin, a macrolide derived from *Streptomyces avermitilis*, can cause coma, delirium, respiratory failure, hypotension, and acidosis. However, certain adverse toxicological effects and psychological implications remain unrecognized in patients undergoing parasuicide.

**Case Presentation:**

Case 1: A 54-year-old man ingested approximately 500 mL avermectin benzylate. He was found to be somnolent 45 min later. Subsequently, the patient became unconscious with respiratory failure, hypotension, and acidosis (lactate, 16.2 mmol/L). Gastric lavage, mechanical ventilation, hemoperfusion, hemofiltration, and symptomatic treatments were administered. Three days after ingestion, the patient’s primary symptoms were delirium and myoclonus, with a blood avermectin level of 110.06 μg/L. Thereafter, intermittent shaking, limb flexion, and muscle rigidity persisted. Thirteen days after ingestion, brain magnetic resonance imaging was unremarkable. Electroencephalography revealed no epileptiform discharges. After psychiatric evaluation, the patient was assessed as having panic-like symptoms, and his symptoms improved significantly with specialized treatment. Case 2: A 33-year-old man was injected with approximately 5 mL of 1% avermectin in his left forearm. Two days later, he reported significant pain and swelling along with intermittent palpitations, dizziness, and limb tremors consistent with panic-like symptoms. His blood avermectin level was 0.02 μg/mL. After 7 days of treatment, the swelling subsided, and the panic-like symptoms resolved.

**Conclusion:**

Avermectin poisoning causes significant toxic effects, including respiratory failure, hypotension, acidosis, coma, and delirium. Intramuscular injections may result in localized swelling. Parasuicide patients may also present with panic-like symptoms requiring careful differential diagnosis.

## 1 Introduction

Avermectins, macrolide compounds derived from *Streptomyces avermitilis*, are widely used as acaricides, insecticides, and nematicides attributed to their cost-effectiveness and low mammalian toxicity (Sung et al., 2009; Wu et al., 2022). Concurrently, deliberate self-poisoning with pesticides has emerged as a significant public health challenge driven by psychosocial factors, a risk amplified by the widespread availability of these agents. Avermectin-induced neurotoxicity is common and typically results in neurological and psychiatric symptoms (Xing, 2020; Wu et al., 2022). However, panic-like symptoms in the context of avermectin poisoning have not been reported previously. This study reports two cases of avermectin poisoning with severe toxic effects and panic-like symptoms, underscoring the need for differential diagnosis.

## 2 Case description

### 2.1 Case one

A 54-year-old man with a history of dysthymia ingested approximately 500 mL avermectin benzylate. He was somnolent when discovered 45 min after ingestion. The patient vomited after waking up. One hour after ingestion, the patient was transferred to a local hospital. Gastric lavage, rehydration, diuresis, and symptomatic treatments were administered. Three hours after ingestion, the patient lost consciousness. Four hours after ingestion, he was transferred to a municipal hospital, where he developed hypotension and vasoactive agents were continuously administered during transport. On admission, the patient was unconscious with a Glasgow Coma Scale score of E1M2V3. His vital signs were as follows: temperature, 36.9 °C; heart rate (HR), 118 bpm; respiratory rate (RR), 10 bpm; blood pressure (BP), 91/86 mmHg; and arterial peripheral oxygen saturation, 85%. A physical examination revealed tachycardia, weak breathing, and hypoactive tendon reflexes. Endotracheal intubation and mechanical ventilation were performed immediately. Subsequent laboratory tests revealed the following: arterial blood gas analysis, FiO_2_ 50%; pH, 7.12; PCO_2_, 30 mmHg; PO_2_, 138 mmHg; lactate, 16.2 mmol/L; WBC, 25.35 × 10^9^/L; hemoglobin, 163 g/L; urea nitrogen, 6.99 mmol/L; and creatinine, 137 μmol/L. Liver function, myocardial enzymes, cholinesterase, coagulation, and electrolyte levels were normal. Hemoperfusion (treatment time: 4 h) and continuous venovenous hemofiltration (CVVH; treatment time: 12 h) were performed. Other treatments included mannitol (oral), proton pump inhibitors, anti-infection drugs, liver protection, hydration, diuresis, and symptom management. The patient remained unconscious even after CVVH. His lactate levels gradually returned to normal. Emergency Computed Tomography (CT) revealed leukoaraiosis and partial shallowing of the brain sulci. Two days after ingestion, his vital signs stabilized after discontinuation of vasoactive agents; however, he began to experience delirium. Three days after ingestion, the patient remained delirious and myoclonic. Blood toxicology revealed that the avermectin level was 110.06 μg/L with negative results for organophosphorus compounds and other pesticides. Subsequently, hemoperfusion was re-administered. However, owing to uncontrolled delirium and myoclonus, he was transferred to Qilu Hospital of Shandong University 5 days post-ingestion. Upon admission, the patient was sedated and vital signs were stable. His blood avermectin level was 0.15 μg/mL with slight elevations in aspartate aminotransferase (85 U/L, normal: 17–59 U/L) and alanine aminotransferase (66 IU/L, normal: 0–50 U/L). Other test results, including routine blood tests, coagulation, renal function, myocardial enzymes, amylase, lipase, cholinesterase, and electrolyte levels were normal. Treatments included sedation, anti-infection, nerve nourishment, hepatoprotection, and symptom management. He intermittently displayed signs of shaking, flexed limbs, and increased muscle tone, accompanied by hypertension and tachycardia, which were relieved following intensive sedation. Seven days after ingestion, ventilation was discontinued and the patient was extubated the next day. Nine days after ingestion, emergency CT revealed mild pneumonia and normal brain function. The patient experienced frequent trismus, limb flexion, muscle rigidity, hypertension, and tachycardia, all of which were alleviated with sedation and sleep. Thirteen days post-ingestion, his prolactin level was 414.6 uIU/L (normal, 86–324 uIU/L), progesterone was 0.980 nmol/L (normal, <0.474 nmol/L), estradiol was 207.4 pmol/L (normal, 41.1–159 pmol/L); cranial magnetic resonance imaging (MRI) showed no abnormalities. Psychiatric evaluation was performed, and the patient was assessed as having panic-like symptoms. Olanzapine and venlafaxine were prescribed. Fifteen days after ingestion, the patient’s psychiatric symptoms improved. He was transferred to a local psychiatric hospital, where his symptoms significantly diminished during transit. Subsequently, electroencephalography was performed and revealed no epileptiform discharges. During follow-up visits, the management of his panic-like symptoms was satisfactory. The clinical course timeline is shown in Figure 1.

**FIGURE 1:**
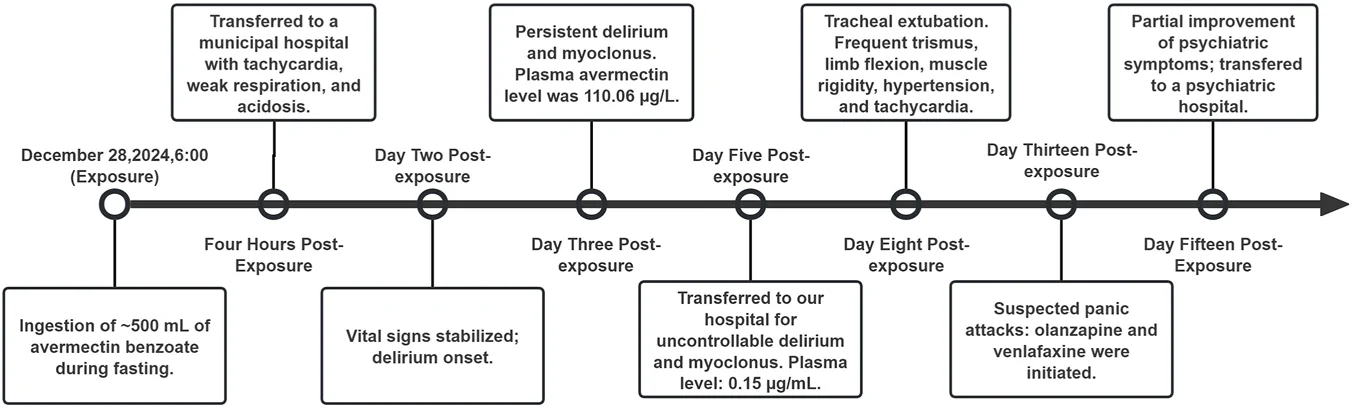
Timeline of clinical course following avermectin benzoate ingestion.

### 2.2 Case two

A 33-year-old man was injected with approximately 5 mL of 1% avermectin into the left medial forearm. The patient initially reported mild pain and did not undergo any interventions. Fifteen hours later, the patient’s left forearm was slightly swollen and painful. Two days later, the left forearm pain and swelling worsened, accompanied by palpitations, dizziness, limb tremors, and panic. The patient informed his family and was admitted to our hospital 50 h after injection. He had a 10-year history of depression and was taking risperidone, valproate sodium, and quetiapine fumarate regularly. On examination, the patient was conscious and cooperative. His vital signs were as follows: temperature, 36.6 °C; HR, 117 bpm; RR, 14 bpm; and BP, 142/92 mmHg. The left forearm was swollen (left circumference, 32 cm; right circumference, 29.5 cm), erythematous, and sufficiently warm to the touch (Figure 2). Other physical findings were unremarkable except for limb tremors. Laboratory results indicated elevated WBC (12.83 × 10^9^/L), neutrophils (81.3%), creatine kinase (265 IU/L), indirect bilirubin (24 μmol/L), direct bilirubin (6 μmol/L), fibrinogen (4.51 g/L), interleukin-6 (84.9 pg/mL, normal 0–7 pg/mL), and tumor necrosis factor-alpha (14.1 pg/mL, normal 0–8.1 pg/mL). Prolactin levels (372 μIU/L, normal 86–324 IU/L) were elevated, and testosterone levels (3.09 nmol/L, normal 6.68–29 nmol/L) were decreased. Toxicology results revealed a blood avermectin concentration of 0.02 μg/mL; other insecticides were undetectable. Renal function, myocardial enzyme levels, electrolyte levels, and thyroid function were within normal limits. Brain MRI showed no abnormalities. Treatments included phosphatidylcholine, magnesium isoglycyrrhizinate, betamethasone, and furosemide. After admission, the patient experienced two episodes characterized by limb tremors, tachycardia, chest tightness, and panic, each lasting approximately 30 min and gradually resolving with reassurance. Psychiatric evaluation was performed, and the patient was assessed as having panic-like symptoms. The day after admission, swelling of the left forearm was reduced, with a circumference of 31 cm. On the third day after admission, the circumference of the left forearm returned to normal, with some hardening at the injection site. As the swelling and pain subsided, his panic-like symptoms improved significantly. After 7 days of treatment, the swelling completely resolved and the patient was discharged. Follow-up 1 week later revealed no significant abnormalities. The clinical course timeline is shown in Figure 3.

**FIGURE 2:**
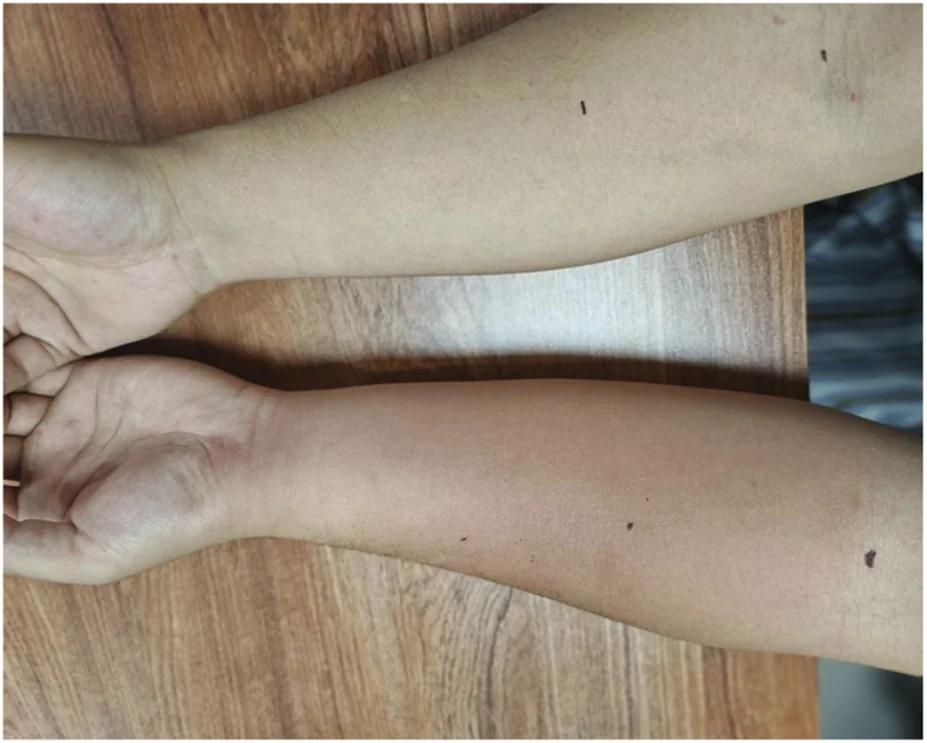
Case 2: 50 hours after intramuscular injection, the swelling in the left forearm is apparent, along with redness.

**FIGURE 3:**
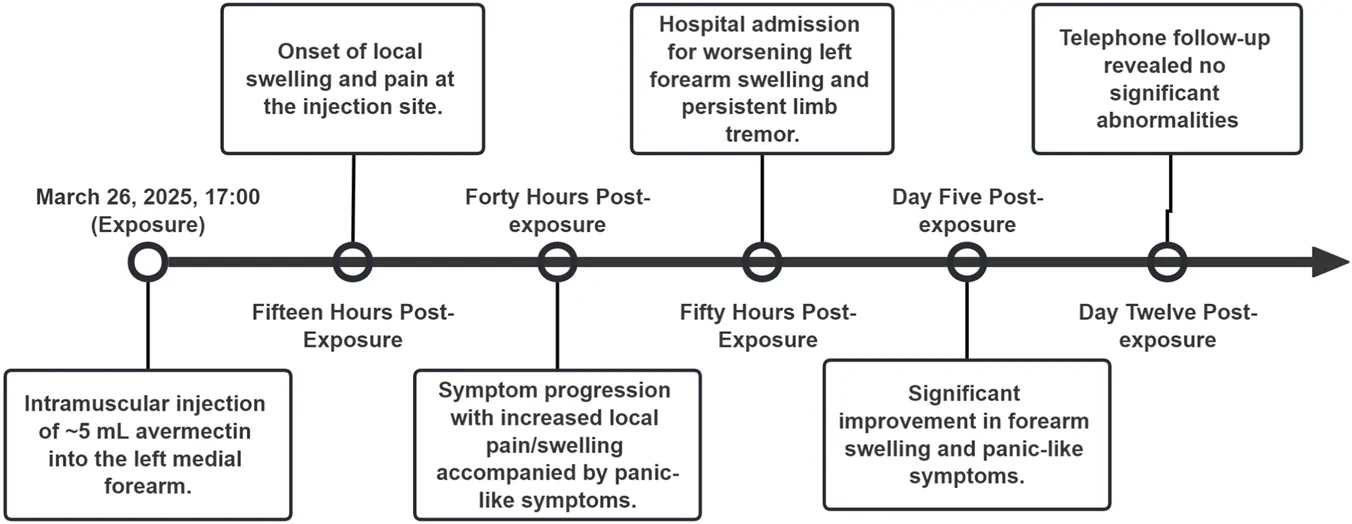
Timeline of clinical course following intramuscular avermectin injection.

## 3 Discussion

Avermectins are a group of macrolide compounds first isolated in 1977 from the bacterium *Streptomyces avermitilis*. These compounds comprise eight fractions: A1a, A1b, A2a, A2b, B1a, B1b, B2a, and B2b, with B1a exhibiting the highest biological activity (Osman et al., 2020). Abamectin is a mixture of 80% avermectin B1a and 20% avermectin B1b and has similar biological and toxicological properties (Lalmalsawmi et al., 2024). Avermectins can interact with invertebrate glutamate-gated chloride channels and gamma-aminobutyric acid (GABA) receptors, inhibiting neurotransmission, and ultimately leading to paralysis and death. These channels are highly expressed in the nerve and muscle cells of nematodes and arthropods but are absent in vertebrate species, except in the central nervous system (CNS). In humans, mild poisoning is often self-limiting and characterized by transient CNS and gastrointestinal symptoms (Wu et al., 2022). This may be related to reduced GABA receptor affinity and the relative impermeability of the blood-brain barrier (Yen and Lin, 2004). However, higher doses of avermectins can cause severe complications such as respiratory failure, hypotension, neurological dysfunction, seizures, coma, or death (Xing, 2020; Wu et al., 2022). CNS abnormalities include altered mental status, confusion, lethargy, agitation, seizures, and coma (Chung et al., 1999; Zhang et al., 2022; Wu et al., 2022). Case 1 developed respiratory failure, hypotension, acidosis, coma, and delirium, which may reflect the direct toxic effects of avermectin. In Case 2, blood avermectin levels remained detectable at 0.02 μg/mL even after 50 h, suggesting ongoing local absorption. Although the patient did not exhibit systemic toxicity, delayed local swelling and pain were observed. Avermectin-induced neurotoxicity may be associated with its effects on various amino acid neurotransmitters and their receptors (Chen et al., 2014). Furthermore, animal studies have demonstrated that avermectin causes histological damage to the cerebrum, cerebellum, and optic lobe of the king pigeon brain (Chen et al., 2013). In addition, autopsy results have revealed widening of the spaces around the cerebral vessels and neural cells in the brain (Dai et al., 2022).

Panic disorder is an anxiety disorder characterized by recurrent panic attacks that involve severe physical symptoms, such as palpitations, sweating, dyspnea, light-headedness, nausea, and trembling, and is often accompanied by intense fear, feelings of losing control, derealization, and significant anxiety about death (Oliva et al., 2021; You et al., 2024; Sasaki et al., 2025). Symptoms may manifest as distinct subtypes that reflect respiratory, cardiovascular, or gastrointestinal issues (Zugliani et al., 2015). Panic disorders are associated primarily with dysfunctional fear networks. The “neuroanatomical hypothesis of panic disorders” specifically highlights this dysfunctional network, which includes the amygdala, hippocampus, medial prefrontal cortex, brainstem nuclei, and the hypothalamic-pituitary-adrenal axis (Kim et al., 2024; Sasaki et al., 2024). Some studies have suggested that fear-related regions tend to be smaller and are accompanied by abnormalities in endocrine hormones (Kim et al., 2024) and amino acid metabolism (such as phenylalanine and tryptophan metabolism) (Shan et al., 2023). In our patients, we found elevated levels of estrogen (progesterone and estradiol) and reduced testosterone levels, which are related to antidepressant use and endocrine abnormalities. P-glycoprotein, derived from *ABCB1* (originally termed *MDR1*), is a substrate transporter that limits avermectin penetration into the CNS by decreasing its influx and increasing its efflux across the blood-brain barrier. Mutations involving *ABCB1* may lead to an increased sensitivity to macrocyclic lactones (Parton et al., 2012). Studies have indicated that *ABCB1* is associated with major depressive disorder and can affect responses to antidepressants (Dong et al., 2009). Sung et al. (2009) reported a 76-year-old man with depression who ingested 200 mL of avermectin and subsequently developed spontaneous generalized myoclonus that resolved on the eighth day of hospitalization. Unfortunately, *ABCB1* genetic testing was not performed in this case or the present study.

Panic-like symptoms, characterized by the abrupt onset of intense fear and autonomic hyperactivity, clinically mimic a typical panic attack but constitute a nonspecific phenotype often resulting from identifiable physiological, toxic, or psychological insults rather than occurring spontaneously. Both patients described in this report exhibited panic-like symptoms: Case 1 presented with tremors, flexed limbs, increased muscle tone, hypertension, and tachycardia, while Case 2 experienced intermittent dizziness, palpitations, and limb tremors. These symptoms may have been triggered by the exacerbation of underlying psychiatric disorders (often precipitated by environmental or psychological stressors), adverse effects of psychotropic medications, or the direct toxic effects of avermectin. Although CNS manifestations are commonly observed in severe avermectin poisoning, symptomatic overlap can lead to the misdiagnosis of panic disorder or the misinterpretation of symptoms as panic attacks, potentially obscuring conditions such as toxic encephalopathy, epilepsy (Kim et al., 2023), delirium, or cardiopulmonary disorders (Hussain and Shah, 2024), particularly because toxic encephalopathy can closely mimic panic-like symptoms. Improvements in panic-like symptoms are frequently associated with cognitive shifts and abrupt symptomatic relief (Lee et al., 2024). In Case 2, significant symptom improvement occurred during the return trip from the hospital, which was likely attributable to leaving the trauma-associated environment. Persistent panic-like symptoms during recovery can compromise treatment compliance and prolong hospitalization. Through this case series, we highlight that psychiatric management is as essential as supportive care in avermectin poisoning. While a causal relationship between avermectin exposure and these symptoms has not been established, we advocate for active monitoring of these psychological sequelae and the adoption of an integrated psychiatric-medical approach in similar cases.

No specific antidote exists for avermectin poisoning and its management primarily focuses on supportive measures. Following oral ingestion, avermectin exhibits limited systemic absorption, attaining peak plasma levels within approximately 3 hours while potentially persisting in the body for a prolonged time (Dai et al., 2022). Regarding Case 1, we noted the apparent increase in levels from Day 3 (110.06 μg/L) to Day 5 (150 μg/L). Following consultation with the testing laboratory, this discrepancy was attributed to variations in detection methods between assays, rather than a true increase in concentration. Fatal cases are frequently caused by respiratory failure, often necessitating aggressive interventions such as tracheal intubation and mechanical ventilation. Therefore, early gastrointestinal decontamination and proactive supportive care, including respiratory support, are essential to improve outcomes.Although empirical hemoperfusion was performed in Case 1 as a locally determined measure, HP is not routinely recommended for avermectin poisoning given its pharmacokinetic profile.

Panic disorders are associated with an elevated suicide risk (Tsai et al., 2025). Furthermore, toxic agents can trigger or exacerbate psychiatric symptoms. In our cases, panic-like symptoms were a prominent feature complicating diagnosis and treatment. Patients with such symptoms benefit from timely psychiatric assessment and multimodal management (Kim, 2019).

## Data Availability

The original contributions presented in the study are included in the article/supplementary material, further inquiries can be directed to the corresponding authors.
